# Development and performance evaluation of slip-resistant agricultural work shoes

**DOI:** 10.1186/1757-1146-7-S1-A109

**Published:** 2014-04-08

**Authors:** Kyung-suk Lee, Young-soon Oh, Do-hee Kim, Hye-seon Chae, Kyung-ran Kim

**Affiliations:** 1 National Academy of Agricultural Science, Rural Development Administration, Suwon, 441-707, Korea

## 

Recently, the burden of farm work and the occurrence of negligent accident increase with aging and feminization in rural areas in South Korea. Especially, accidents like slip or falling occur as the most frequent farm work accidents, and a half of female farmers have experienced an accident by slip. The Korea’s agricultural environment is poor with bumpy ground and slippery ground with water, which may cause farmers get a heavy load on the feet: e.g. squatting, and accordingly deformation or damage to the foot is frequently observed, but there are no development and research on agricultural work shoes to improve the problems. Thus, this study develops agricultural work shoes with enhanced slip-resistant performance and attempts to look into the performance of the developed agricultural work shoes through a comparison with existing products.

Agricultural work shoes were produced based on the results of research on the conditions of work shoes put on for dry-field farming, referring to the outsole of mountaineering boots put on in environments most similar to agricultural field, which are light and have a performance similar to that of existing products. They were in a form of 6-inch safety shoes that could prevent twisting of the ankles and a last reflecting morphological characteristic of the farmers’ feet were applied, and a mesh material was used to enhance their thermal comfort.

For a performance evaluation, a slip-resistance evaluation and a gait stability evaluation according to the plantar foot weight dispersion were conducted on 4 existing products and 1 developed product. For the slip-resistance evaluation, an AVIT measurement (detergent solution, glycerin solution) and an HSL ramp test were carried out, and the work shoes used in the test had the irregular roughness of the outsole surface and since the actual slip accidents occurred in old work shoes rather than in new ones, the outsole of the work shoes was brushed with sandpaper 400 times before the test. For the plantar foot pressure evaluation, the distribution of the plantar foot pressure was measured using Novel Padar-x System for two men who had not experienced any pain or illness at the lower part of the body or foot and had a normal gait form.

1. As a result of a slip resistance evaluation, all work shoes including the developed product showed excellent performance in detergent solution while in glycerol, most of them were below Grade 2. It turned out that mountaineering boots had the highest risk of slipping, but the developed agricultural work shoes had a relatively lower risk of slip except in glycerol.

2. As a result of a plantar foot pressure evaluation, the contact area was in the order of mountaineering boots (136.5cm^2^), new model rubber shoes (121.3cm^2^) and development product (120.0cm^2^), and the maximum pressure was relatively higher in mountaineering boots than that of new model rubber shoes and the developed product. The developed agricultural work shoes had a weaker weight dispersion effect compared to new model rubber shoes while they substantially induced a wide contact area and evenly dispersed plantar foot pressure.

Through the above results, the developed agricultural work shoes had a better performance than mountaineering boots put on in similar environments. Yet, it is somewhat impractical to evaluate them as a standard for the performance evaluation of industrial safety shoes with which the main task is carried out on the relatively flat ground, and it is judged that in the future, multifaceted research on performance evaluation specifications and test methods of work shoes suitable for the farm work environment will be necessary.

